# Evaluation of Two Different Methods of Fulvic Acid Application (Seed Priming and Foliar Spray) on Growth, Yield, and Nutritional Quality of Pea (*Pisum sativum* L.)

**DOI:** 10.3390/plants13233380

**Published:** 2024-11-30

**Authors:** Rehab M. Mahdy, Adel M. Al-Saif, Mohamed E. M. Ahmed, Tahany S. Abd El-Bary, Ashutosh Sharma, Abdel-Nasser A. El-Sheshtawy, Rasha S. El-Serafy, Tamer S. Abd El-Ghany

**Affiliations:** 1 Horticulture Department, Faculty of Agriculture, Tanta University, Tanta 31527, Egypt; dr.memahmed@agr.tanta.edu.eg; 2 Department of Plant Production, College of Food and Agriculture Sciences, King Saud University, P.O. Box 2460, Riyadh 11451, Saudi Arabia; adelsaif@ksu.edu.sa; 3 Potato and Vegetatively Propagated Vegetables Department, Horticulture Research Institute, Agriculture Research Center, Giza 3725005, Egypt; tahanyshaaban53@gmail.com; 4 Faculty of Agricultural Sciences, DAV University, Jalandhar 144012, Punjab, India; ashutosh10110@davuniversity.org; 5 Environment and Bio-Agriculture Department, Faculty of Agriculture, Al-Azhar University, Cairo 11884, Egypt; abdel_nasser2007@azhar.edu.eg (A.-N.A.E.-S.); tamer_said2013@azhar.edu.eg (T.S.A.E.-G.)

**Keywords:** fulvic acid, seed priming, foliar spray, carbohydrates, protein, antioxidant enzymes

## Abstract

Pea is a commercially significant legume that is widely utilized worldwide and has a elevated amount of nutrition and bioactive components. Its consumption is attributed to a number of health benefits and its potential as a functional food. Fulvic acid can be used as a bio-stimulant to promote plant growth and increase nutrient availability and uptake. A field experiment was designed during two subsequent cropping seasons (i.e., 2022–23 and 2023–24) to evaluate the impact of two methods of fulvic acid application of seed priming and foliar spray on the growth, yield, antioxidant content, and nutritional value of pea (*Pisum sativum* L.) plants. The seeds were primed with fulvic acid at 1.5, 3 g L^−1^, and water, while a foliar spray of fulvic acid with the same doses was performed on the seedlings of non-primed seeds. The results obtained exhibited that the seed priming technique with fulvic acid outperformed the fulvic acid foliar applications. The increase in the fulvic acid dose to 3 g L^−1^ in both application techniques exhibited the highest plant growth, heaviest fresh and dry weights, and highest green seed yield. The maximum growth parameters were recorded after the foliar spray treatment at a dose of 3 g L^−1^, as it led to improvement in the growth parameters, leaf pigments, and total carbohydrates. The highest number of seeds per pod, number of pods per plant, 100-seed weight, and seed yield were obtained by the seed priming technique. From the results obtained, it may be concluded that the application of fulvic acid at 3 g L^−1^ via the seed priming technique is beneficial for enhancing the productivity of peas.

## 1. Introduction

*Pisum sativum* L. (pea) is a popular, valuable legume crop, cultivated widely all over the world and considered an essential component of the human diet [1,2]. It contains protein, complex carbohydrates, bioactive substances, minerals, and vitamins that are important for human health and nutrition [1,2,3]. Complex carbohydrates are the major components of pea seeds (59.32–69.59%) [4]. Pea proteins (20–25% of the dry matter) and peptides have a variety of biological activities [5,6], including metabolic syndrome control. Peas are also high in dietary fiber (23–31%) [7], which has a variety of health advantages via modulating gut microbiota [8]. Pea seeds are preferably consumed by people who have coeliac disease because they are gluten-free [9]. In addition, it includes a variety of minerals and vitamins, including folic acid and carotenoids [10], as well as a high concentration of polyphenolics, particularly flavonoids, which have a variety of biological functions [1,2,3]. The chemical attributes and functional characteristics of pea seeds are dependent on the cultivar, environmental conditions, and agricultural practices.

Despite the effectiveness of chemical fertilizers for improving agricultural productivity, they pollute the air and groundwater while also having a significant impact on climate change [1,11,12]. Further, agroecosystem conservation and improvement in plant productivity are key concerns [13,14,15]; thus, it is vital to reduce the dependence on conventional agricultural practices with the increased deployment of more secure ones [16,17,18]. Numerous attempts have been made to minimize the dependency on synthetic fertilizers by employing bio-stimulants as their alternative. Using bio-stimulants is advised as an uncommon technique to improve the sustainability of crops and eliminate the use of artificial fertilizers [19,20,21].

Fulvic acid, an organic bio-stimulant, is a non-toxic mineral-chelating addition and water binder that improves nutrient absorption and increases plant production [22]. It binds water molecules, keeping the soil wet and facilitating the transfer of nutrients to plant roots. Fulvic acid readily chelates minerals such as Fe, Ca, Zn, and Mg, which it may transfer straight to plants [23]. Sun et al. [24] revealed that fulvic acid maximized the P availability in the soil, while Mahmoud et al. [25] recorded that onion plants subjected to fulvic acid treatments exhibited the best growth, yield, and bulb quality. Fulvic acid application at the dose of 3 g L^−1^ to two cultivars of okra was the ideal dose for giving higher growth, fruit yield, and nutritional value [26]. Moreover, Nadia et al. [27] reported that fulvic acid application produced the highest plant growth, total yield, average tuber weight, and dry matter of *Helianthus tuberosus* L.

Seed-priming is a pre-sowing technique of seed preparation that permits the water to penetrate the seeds and starts the first stage of seed germination while preventing radical emergence from the seed coat [28]. Seed-priming treatments stimulated the plant height, seedling biomass, leaf pigments, and total carbohydrates of *Lathyrus odoratus* under salinity stress [29]. Seed priming treatments may stimulate the growth performance of pea plants both under normal as well as stressful conditions [30]. At the same time, foliar application is a method that provides important nutrients to plants by spraying a water-based solution effectively onto the foliage, which receives nutrients through its cuticles and stomata [31,32]. Foliar spraying can be beneficial when plants need a particular nutrient, but it is not an alternative for good soil. Furthermore, foliar spraying treatment prevents nutrient leakage and stimulates a quick reaction by the plant [33]. Wang et al. [34] reported that exogenous foliar spraying with fulvic acid improved lettuce growth and increased plants’ tolerance to environmental stress. In addition, Suhŏ et al. [35] reported that tomato plants exposed to fulvic acid foliar spraying experienced a promotion of their growth and marketable value. Under stress conditions, seed priming with different bio-stimulants led to an improvement in the growth, seed yield, and nutritional value of pea plants [36]; therefore, the current investigation was layout to evaluate the seed priming and foliar treatment of fulvic acid to improve plant growth, metabolites, quality, and yield in pea. Further, a comparison between two different modes of fulvic acid applications (i.e., seed priming and foliar spraying) was made on the growth, green seed yield, and nutritional value in order to find out their relative improvement of the above traits. To the best of our knowledge, this is the first report of the comparison of two different modes of application of fulvic acid as a bio-stimulant in peas.

## 2. Materials and Methods

### 2.1. Experimental Site, Soil Preparation and Analysis

In a private nursery farm in El-Santa District, Algharbia Governorate, Egypt (30°50′56″ N 31°06′49″ E), two field investigations were performed during the winter seasons of 2022/2023 and 2023/2024. The experimental field was divided into different plots; each plot area was 12 m^2^, consisting of 4 rows. The length and width of each row were 5 m and 0.6 m, respectively, with a plant spacing of 10 cm. Before sowing, the soil parameters were analyzed according to Page et al. [37] and Klut [38] methods. The soil type was clay loamy, and the results of soil analyses were as follows: 41.7% silt, 34.5% clay, and 23.8% sand, pH; 7.16, EC; 0.50 dS m^−1,^ total N, P, and K; 48.0,6.08, and 640 ppm, in respective, Mg; 1.0 meq L^−1^, Ca^2+^; 185.4 meq L^−1^, HCO_3_^−1^; 2.0 meq L^−1^, SO_4_; 0.5 meq L^−1^.

### 2.2. Plant Materials and Seed Priming Treatments

The uniform and fully mature pea seeds (*Pisum sativum* L., Hende) were surface sterilized using a 10% sodium hypochlorite (NaClO) solution (*v*/*v*) for 5 min, followed by the washing of the sterilized seeds using distilled water thrice. The seeds were then divided into three groups for seed priming treatments for 10 h; the first group was primed with fulvic acid solution at 1.5 g L^−1^ (SPF1), the second was primed with 3 g L^−1^ of fulvic acid solution (SPF2), and the third was primed with distilled water (SPW). After that, the primed seeds were air-dried and prepared to be sown in the field.

### 2.3. Foliar Spray Treatments

All primed and non-primed seeds were sown in plots on the 11 and 15 of November for the two growing seasons, respectively. After 40 days, the seedlings were thinned to one plant/hill, and the non-primed-seeds foliage was sprayed with different doses of fulvic acid as follows: (1) 1.5 g L^−1^ (FSF1), (2) 3 g L^−1^ (FSF2), and (3) tap water (FSW). The foliar applications were performed thrice; the first one was applied 40 days after sowing, and every 15 days from each application. The two experiments were designed in a randomized complete block design with six treatments, each treatment replicated three times (each replication performed in one plot). All agricultural practices were performed as recommended.

### 2.4. Growth Traits and Yield Performance

At the harvesting stage, ten plants from each replicate were randomly selected for plant height (cm), leaf number plant^−1^, and herb fresh and dry weight plant^−1^ assessment. Yield-related traits of the number of pods plant^−1^, pod length, number of seed pod^−1^, 100-seed weight, seed yield plant^−1^, and green seed yield ha^−1^ were estimated.

### 2.5. Physio-Biochemical Analysis

#### 2.5.1. Leaf Pigments

At the flowering stage, the leaf samples were collected for biochemical analysis. The leaf pigment contents of chlorophyll A, B, carotenoids, and total chlorophyll were estimated following the methods of Dere [39] and presented in mg g^−1^ FW.

#### 2.5.2. Total Phenols and Antioxidant Enzyme Activity

The total phenol content (mg g^−1^) was assessed spectrophotometrically following the Folin-Ciocalteu’s reagent method with Gallic acid as the quantification standard as McDonald et al. [28] procedure. The antioxidant enzyme activities (mg g^−1^) were measured spectrophotometrically in the supernatant of fresh leaves. Catalase (CAT) activity was measured according to Aebi [40], and peroxidase (POX) activity was measured in the crude enzyme extract according to Hammerschmidt et al. [41]. Polyphenol oxidase (PPO) activity was estimated using the Malik and Singh [42] methods.

#### 2.5.3. Total Carbohydrates and Protein

Total carbohydrates (%) in the pea seeds were determined using the colorimetric method of Dubois et al. [43]. The total soluble protein (%) in the seeds was measured using the Folin-Ciocalteu reagent [44].

#### 2.5.4. Nutrient Estimation

Nutrient estimation was performed on the dried seeds. Total nitrogen (N) was determined using the nitrogen-to-protein conversion factor (6.25) [45], phosphorus (P) was measured by Watanabe and Olsen [46], while potassium (K) was measured using Flam photometer according to Brown and Lilliland [47].

### 2.6. Statistical Analysis

The results presented in tables and figures are the mean ± SE (standard error) of the independent replicates. The significant difference among the treatments was testing using COSTAT version 6.4 software at *p* ≤ 0.05. Duncan’s multiple range test was employed to estimate the significant difference between the mean values of the treatments (*p* ≤ 0.05).

## 3. Results

### 3.1. Growth Performance

Growth traits presented in Table 1 and Table 2 exhibited that pea plants subjected to foliar spray showed an enhancement in their values relative to the seed priming technique. Plant height, number of leaves, fresh weight, and dry weight of foliar-sprayed plants with 3 g L^−1^ exhibited the highest values of 106.3 cm, 20.3, 34.9 g, and 9.8 g for the first and 110.7 cm, 26.7, 36.5 g, and 9.8 g for the second seasons, respectively. The least growth values were shown by pea plants that were foliar sprayed with water, giving 86.3 cm, 12.0, 18.2 g, and 6.9 g for the first and 88.3 cm, 15.7, 20.3 g, and 7.1 g for the second seasons, respectively. These results suggest that applying fulvic acid through foliar spraying at 3 g L^−1^ is effective in promoting vegetative growth, aligning with this study’s goal of identifying the best method to maximize growth parameters.

### 3.2. Yield Performance

**Figure 1:**
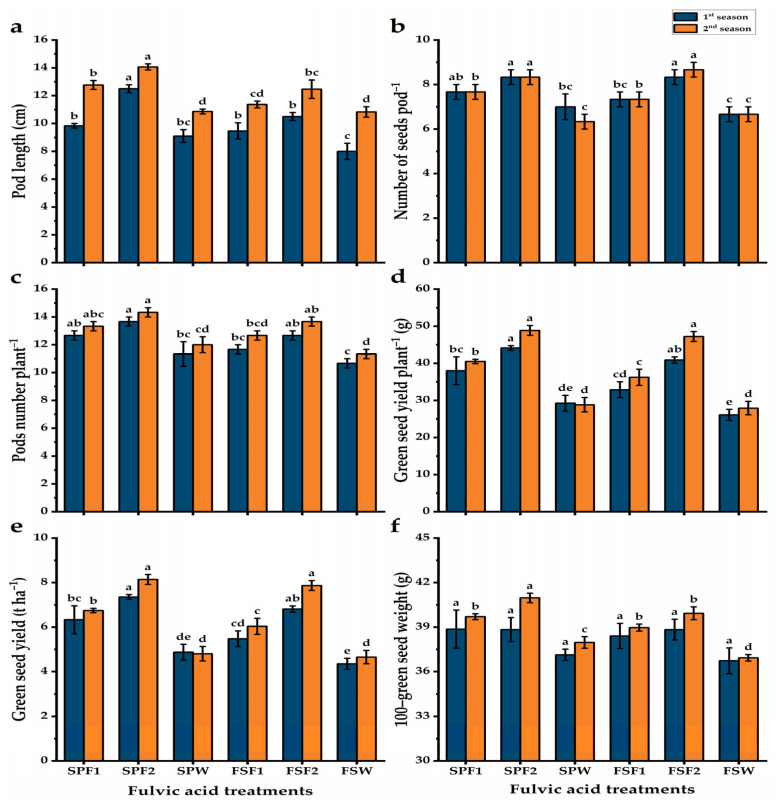
Pod length (**a**), number of seeds pod^−1^, (**b**), pods number plant^−1^ (**c**), green seed yield plant^−1^ (**d**), green seed yield ha^−1^ (**e**), 100-green seed weight (**f**) of pea plants in response to two methods of fulvic acid additions during two agricultural seasons. Data are represented as mean value ± SE. Means with different letters on the bars (a–d) for each season significantly differed, using Duncan’s multiple range test at *p* ≤ 0.05. SPF1; seed priming with 1.5 g L^−1^ fulvic acid, SPF2; seed priming with 3 g L^−1^ fulvic acid, SPW; seed priming with distilled water, FSF1; foliar spray with 1.5 g L^−1^, FSF2; foliar spray with 3 g L^−1^ fulvic acid, and FSW; foliar spray with tap water.

Yield and yield-related traits mean values are presented in Figure 1. Pea plants whose seeds were primed showed significant improvement in the yield contributing traits relative to the foliar spray technique. The highest values of pod length, number of pods plant^−1^, seed pod^−1^, and 100-seed weight were obtained by seed-primed plants with 3 g L^−1^, as well as these plants showed a growth in the green seed yield plant^−1^ by about 69 and 75% higher than those plants which were foliar sprayed with water for both seasons, respectively. While, the lowest values were exhibited by the pea plants, which received the foliar spray with water. The enhancement in seed yield following 3 g L^−1^ of fulvic acid by the foliar spray technique emphasizes the goal of this study.

### 3.3. Leaf Pigments Content

The pigment content in pea leaves in response to fulvic acid supplementation is presented in Table 3. The foliar spray application at 3 g L^−1^ considerably outperformed the priming treatments for chlorophyll A, B, total, and carotenoids content by 3.8, 2.42, 6.27, and 1.98 mg g^−1^ FW for the first season and 3.9, 2.9, 6.91, and 2.1 mg g^−1^ FW for the second season, respectively. While seed priming with fulvic acid at 3 g L^−1^ ranked second in terms of chlorophyll A, B, total, and carotenoids (3.5, 1.9, 5.53, and 1.4 mg g^−1^ FW for the first season and 3.6, 2.3, 5.8, and 1.6 mg g^−1^ FW for the second, respectively). In contrast, water applications achieved the lowest leaf pigment content in pea leaves.

### 3.4. Total Phenols and Antioxidant Enzyme Activities

The total phenolics and antioxidant enzyme activities of pea seeds were both considerably raised by all fulvic acid doses and both application methods as compared with water treatments (Table 4). In comparison to the rest of the treatments, the seed priming at 3 g L^−1^ fulvic acid treatment substantially (*p* ≤ 0.01) gave higher total phenols, CAT, POX, and PPO, as recorded 0.95 and 1.06 mg g^−1^ for total phenols, 1.45 and 1.75 µmol min^−1^ g^−1^ for CAT, 1.79 and 2.16 U g^−1^ for POX, and 0.7 and 1.18 U g^−1^ for PPO enzyme for the two seasons, respectively. The lowest values recorded were, however, exhibited by pea plants that were treated with water foliar spray.

#### 3.4.1. Total Carbohydrates and Protein in Seeds

**Figure 2:**
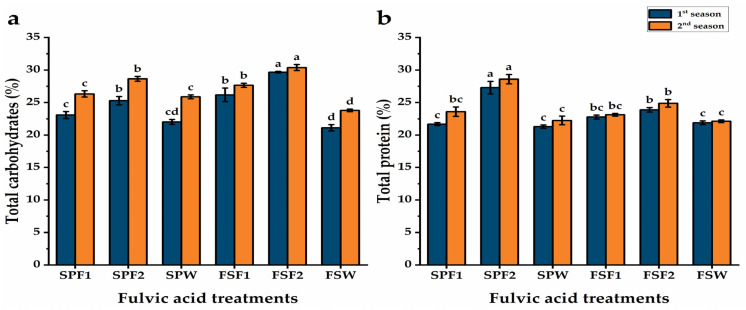
Total carbohydrates (%) (**a**) and total protein (%) (**b**) of pea seeds in response to two methods of fulvic acid additions during two agricultural seasons. Data are represented as mean value ± SE. Means with different letters on the bars (a–d) for each season significantly differed, using Duncan’s multiple range test at *p* ≤ 0.05. SPF1; seed priming with 1.5 g L^−1^ fulvic acid, SPF2; seed priming with 3 g L^−1^ fulvic acid, SPW; seed priming with distilled water, FSF1; foliar spray with 1.5 g L^−1^, FSF2; foliar spray with 3 g L^−1^ fulvic acid, and FSW; foliar spray with tap water.

The total carbohydrates and protein content of pea seeds were raised by all fulvic acid doses and both application methods as compared with water treatments (Figure 2). In comparison to the rest of the treatments, the foliar spray at 3 g L^−1^ fulvic acid treatment substantially (*p* ≤ 0.01) increased total carbohydrates by about 40 and 27.6% higher for the two seasons, respectively. At the same time, the plants with seed priming treatment with the same dose of fulvic acid showed the second rank in this respect. Regarding protein content, the seed priming technique was found to be more effective in improving protein content than foliar spraying and using the highest fulvic acid level recorded the highest protein content for both seasons.

#### 3.4.2. Nutrient Content

The results depicted in Table 5 revealed that all fulvic acid applications significantly boosted the absorbation of the essential elements of N, P, and K in comparison to the water treatments (Table 5). The mineral content of N, P, and K in pea seeds collected from seed priming at 3 g L^−1^ fulvic acid was substantially greater than the other treatments. At the same time, the minimum nutrient values were obtained by water-foliar-sprayed plants for both seasons.

## 4. Discussion

This experiment was performed to evaluate the effects of two methods of fulvic acid applications (seed priming and foliar spray) on the growth, yield, and nutritional value of pea plants. Despite this, fulvic acid application has been widely reported to stimulate and enhance plant growth of many crops [48,49], but the comparison between the two methods of application and their effects on plant growth is still unclear. The responses of plants to humic substances are highly impacted by the fulvic acid source, the species of the plant and development stage, environmental conditions, application methods, doses, and planting media [50]. The same observation was noticed by Martins et al. [51], who stated that the rates and techniques of applications of fulvic acid have a significant impact on growth promotion. The results obtained in this experiment revealed that fulvic acid treatments increased the plant height, fresh and dry weights, and the number of leaves of pea plants. These findings may be due to the fact that fulvic acid has the function of indole acetic acid (IAA), which increases the cell division and expansion of roots and regulates root growth [50], leading to more water and nutrient absorption and, consequently, plant growth. Bayat et al. [52] evaluated the effect of varying fulvic acid rates on growth traits of *Achillea millefolium* cultivated in sandy loam soil, and they found that all treatments dramatically enhanced both fresh and dry weights in comparison to the untreated control plants. The same authors found that the biggest increases were seen at the highest rates, resulting in a 50% increase in dry weight as compared to control plants. Fulvic acid stimulates nutrient uptake via the chelating agent and boosts vegetative growth, nutritional value, and leaf pigment composition [53].

Humic substances can improve crop quality and productivity by promoting plant development, fruit number, size, and yield [54]. In this study, an increment in the pod number, length, and green seed yield of pea plants was observed following seed priming and foliar application with fulvic acid, and seed priming outperformed foliar application in this regard. Seed priming is a simple and efficient technique that promotes uniform and rapid emergence with more seedling vigor and better plant development, which finally increases the crop yield under different environmental conditions [55,56]. In this study, the bio-stimulating influence of foliar spray on pea growth has been observed during the vegetative stage (plant height, leaf and branch numbers, fresh and dry weights), while at the reproductive stage, the seed priming technique presented more effectiveness than the other application (foliar spraying), and these findings reveal that both these application techniques have different mechanisms of action. Husein et al. [57] revealed that foliar treatment with fulvic acid improved growth, shoot fresh and dry weight, fruit hardness, and yield in field-grown tomatoes. Fulvic acids are important components of excessively strong foliar fertilizers, as they can absorb huge amounts of water and nutrients, also, fulvic acid is rich in C and N elements, the necessary elements for vegetative growth [54]. They might aid in the penetration of plant parts and initiate the absorption of compounds from plant surfaces into the plant tissues [57]. When applied to the foliage, fulvic acids help in transferring essential minerals straight to metabolic areas within the plant cells. So, foliar spray treatments at specific plant boom degrees, including mineral chelated, can be used as a rapid technique for improving plant growth efficiency potential [58].

Fulvic acid may have a positive influence on vegetative development since it improves the availability of cellular antioxidants, phytohormones such as IAA, cytokines, and GA_3_, as well as various vitamins such as vitamin B [59]. Fulvic acid stimulates optimal development and replication of cells while also enhancing cell division and elongation [60]. Fulvic acid may have a favorable effect on chlorophyll content, as it improves both oxygen intake and the concentration of mRNA (Messenger Ribonucleic Acids) in plant cells. Both of them are required for various metabolic activities in plant cells, including enzyme and protein synthesis, which leads to an increase in chlorophyll production [61,62]. Fulvic acid treatments can stimulate the overexpression of the genes involved in important plant metabolic processes, including plant development, plant hormone regulation, photosynthesis, and nitrogen/sulfur metabolism [63]. Fulvic acid significantly promotes the antioxidant capacity of plants by increasing flavonoid biosynthesis, ascorbic acid metabolism, and glutathione metabolism [24]. Our findings revealed that pea plants subjected to fulvic acid treatments led to an increase in the total carbohydrates, total phenols, and protein content. Similar results were noticed by He et al. [64], who reported that the fulvic acid spraying technique led to a rise in the levels of total sugar, vitamin C, total flavonoids, and phenols in lemon fruits. Ren et al. [65] stated that both fulvic acid spraying and irrigation techniques exhibited a positive influence on lemon fruit biochemical analysis components, and irrigation yielded better than spraying.

Fulvic acid treatments elevated N, P, and K content in pea tissues, and the maximum values were obtained by seed priming treatment at 3 g L^−1^. An increase in K concentrations was noticed following fulvic acid treatments [51]. One of the most significant benefits of humic substances for horticultural crop growers would be increased nutrient-use efficiency, which allows them to produce high-quality crops with fewer inorganic fertilizer inputs [66]. It is generally known that humic substances improve nutrient availability and plant uptake. General improvements in nutrient absorption are caused by hormonal-type bio-stimulation of lateral root induction and root hair commencement [50]. In tomato plants, there was an increase in the P and K content in tomato leaves following humic acid supplementation [57]. In addition, an elevation in the N content was observed as affected by fulvic acid applications in yarrow plants [52].

## 5. Conclusions

Fulvic acid supplementation by two methods viz., seed priming and foliar spray, is a potential method of promoting the growth and productivity of pea plants. Fulvic acid enhances growth by improving photosynthetic effectiveness and nutrient homeostasis. Further, the addition of fulvic acid via the two methods may effectively boost seed yield and seed quality as well as the plant’s biochemical composition in terms of maximizing carbohydrate and protein contents, phenolics, and enzymatic activity; therefore, applying fulvic acid application by seed priming or foliar spray would be a sustainable for improving agricultural output. Our findings also revealed that seed priming with fulvic acid can be effectively used to improve plant growth and output of peas.

## Figures and Tables

**Table 1:** Plant height and number of leaves of pea plants in response to various treatments involving two different methods of fulvic acid application (priming and foliar spray).

Treatment	Plant Height (cm)		Number of Leaves	
	1st Season	2nd Season	1st Season	2nd Season
SPF1	94.0 ± 1.15 c	98.3 ± 2.03 bcd	14.00 ± 0.58 bc	20.33 ± 1.45 b
SPF2	97.7 ± 1.86 bc	100.3 ± 6.06 abc	16.00± 0.58 bc	22.00 ±1.53 b
SPW	88.0 ± 1.53 d	90.00 ± 1.15 b	13.00 ± 0.58 c	19.67 ± 1.20 b
FSF1	99.3 ± 1.20 b	104.67 ± 2.91 ab	17.33 ± 2.19 ab	22.33 ± 1.45 b
FSF2	106.3 ± 0.88 a	110.67 ± 2.33 ab	20.33 ±0.88 a	26.67 ± 0.88 a
FSW	86.3 ± 0.88 d	88.33 ± 2.19 b	12.00 ± 1.73 b	15.67 ± 1.20 c

Mean values sharing the same lowercase letter in the same column do not differ significantly at *p* ≤ 0.05 by Duncan’s multiple range test. SPF1; seed priming with 1.5 g L^−1^ fulvic acid, SPF2; seed priming with 3 g L^−1^ fulvic acid, SPW; seed priming with distilled water, FSF1; foliar spray with 1.5 g L^−1^, FSF2; foliar spray with 3 g L^−1^ fulvic acid, and FSW; foliar spray with tap water.

**Table 2:** Fresh and dry weights of pea plants in response to various treatments involving two different methods of fulvic acid application (seed priming and foliar spray).

Treatment	Fresh Weight (g)		Dry Weight (g)	
	1st Season	2nd Season	1st Season	2nd Season
SPF1	23.72 ± 1.10 c	25.89 ± 0.67 c	8.01 ± 0.26 c	8.61 ± 0.20 b
SPF2	28.16 ± 1.10 b	29.83 ± 1.17 b	8.83 ± 0.20 b	9.04 ±0.20 b
SPW	19.67 ± 1.16 d	21.50 ± 0.59 d	7.19 ± 0.29 d	7.25 ± 0.31 c
FSF1	30.93 ± 0.85 ab	33.33 ± 0.46 ab	8.99 ± 0.17 b	9.15 ± 0.07 ab
FSF2	34.87 ± 1.86 a	36.53 ± 1.71 a	9.80 ± 0.21 a	9.87 ± 0.03 a
FSW	18.15 ± 1.10 d	20.32 ± 1.49 d	6.96 ± 0173 d	7.10 ± 0.30 c

Mean values sharing the same lowercase letter in the same column do not differ significantly at *p* ≤ 0.05 by Duncan’s multiple range test. SPF1; seed priming with 1.5 g L^−1^ fulvic acid, SPF2; seed priming with 3 g L^−1^ fulvic acid, SPW; seed priming with distilled water, FSF1; foliar spray with 1.5 g L^−1^, FSF2; foliar spray with 3 g L^−1^ fulvic acid, and FSW; foliar spray with tap water.

**Table 3:** Leaf pigments of pea plant in response to various treatments involving two different methods of fulvic acid application (seed priming and foliar spray).

Treatment	Chlorophyll A (mg g^−1^ FW)		Chlorophyll B (mg g^−1^ FW)		Total Chlorophyll (mg g^−1^ FW)		Carotenoids (mg g^−1^ FW)	
	1st Season	2nd Season	1st Season	2nd Season	1st Season	2nd Season	1st Season	2nd Season
SPF1	3.25 ± 0.03 b	3.39 ± 0.19 ab	1.66 ± 0.07 b	1.79 ± 0.29 bc	4.91 ± 0.1 bc	5.18 ± 0.2 bc	1.23 ± 0.06 bc	1.43 ± 0.15 b
SPF2	3.49 ± 0.07 ab	3.58 ± 0.12 ab	1.93 ± 0.09 ab	2.26 ± 0.16 abc	5.42 ± 0.1 b	5.84 ± 0.3 b	1.38 ± 0.21 bc	1.58 ± 0.05 b
SPW	2.98 ± 0.09 b	3.01 ± 0.39 b	1.56 ± 0.26 b	1.62 ± 0.29 bc	4.53 ± 0.3 c	4.63 ± 0.6 c	1.14 ± 0.07 bc	1.34 ± 0.11 b
FSF1	3.29 ± 0.09 b	3.31 ± 0.07 ab	1.88 ± 0.12 ab	2.38 ± 0.10 ab	5.17 ± 0.1 b	5.69 ± 0.1 b	1.43 ± 0.10 b	1.64 ± 0.10 ab
FSF2	3.86 ± 0.29 a	3.95 ± 0.26 a	2.42 ± 0.05 a	2.95 ± 0.04 a	6.27 ± 0.3 a	6.91 ± 0.2 a	1.98 ± 0.09 a	2.08 ± 0.23 a
FSW	2.40 ± 014 c	2.80 ± 0.35 b	1.47 ± 0.26 b	1.57 ± 0.27 b	3.87 ± 0.2 d	4.38 ± 0.1 c	1.06 ± 0.03 c	1.30 ± 0.13 b

Mean values sharing the same lowercase letters (a–d) in the same column do not differ significantly at *p* ≤ 0.05 by Duncan’s multiple range test. SPF1; seed priming with 1.5 g L^−1^ fulvic acid, SPF2; seed priming with 3 g L^−1^ fulvic acid, SPW; seed priming with distilled water, FSF1; foliar spray with 1.5 g L^−1^, FSF2; foliar spray with 3 g L^−1^ fulvic acid, and FSW; foliar spray with tap water.

**Table 4:** Total phenols and antioxidant enzyme activity of pea leaves in response to various treatments involving two different methods of fulvic acid application (seed priming and foliar spray).

Treatments	Total Phenols (mg g^−1^)		CAT (µmol min^−1^ g^−1^)		POX (U g^−1^)		PPO (U g^−1^)	
	1st Season	2nd Season	1st Season	2nd Season	1st Season	2nd Season	1st Season	2nd Season
SPF1	0.65 ±0.05 bc	0.75 ± 0.01 b	0.93 ± 0.14 bcd	1.06 ± 0.06 cd	1.04 ± 0.02 c	1.64 ± 0.05 a	0.50 ± 0.02 c	0.71 ± 0.15 b
SPF2	0.95 ± 0.03 a	1.06 ± 0.11 a	1.45 ± 0.04 a	1.75 ± 0.10 a	1.79 ± 0.07 a	2.16 ± 0.41 a	0.70 ± 0.02 a	1.18 ± 0.24 a
SPW	0.54 ± 0.08 cd	0.63 ± 0.26 bc	0.63 ± 0.09 d	0.85 ± 0.09 d	0.83 ± 0.04 d	0.98 ± 0.01 b	0.37 ± 0.01 d	0.73 ± 0.04 b
FSF1	0.69 ± 0.01 bc	0.73 ± 0.06 b	1.01 ± 0.11 bc	1.14 ± 0.05 c	1.10 ± 0.02 c	1.63 ± 0.05 a	0.55 ± 0.05 bc	0.79 ± 0.07 b
FSF2	0.76 ± 0.03 b	0.86 ± 0.08 b	1.13 ± 0.08 b	1.44 ± 0.06 b	1.32 ± 0.05 b	1.86 ± 0.08 a	0.64 ± 0.02 ab	0.86 ± 0.03 ab
FSW	0.49 ± 0.02 d	0.52 ± 0.05 c	0.78 ±0.09 cd	0.95 ±0.04 cd	0.88 ± 0.05 d	0.93 ± 0.01 b	0.29 ± 0.03 d	0.60 ± 0.07 b

Catalase (CAT), Peroxidase (POX), polyphenol oxidase (PPO). Mean values sharing the same lowercase letters (a–d) in the same column do not differ significantly at *p* ≤ 0.05 by Duncan’s multiple range test. SPF1; seed priming with 1.5 g L^−1^ fulvic acid, SPF2; seed priming with 3 g L^−1^ fulvic acid, SPW; seed priming with distilled water, FSF1; foliar spray with 1.5 g L^−1^, FSF2; foliar spray with 3 g L^−1^ fulvic acid, and FSW; foliar spray with tap water.

**Table 5:** Macronutrients content in pea leaves in response to various treatments involving two different methods of fulvic acid application (seed priming and foliar spray).

Treatment	N (%)		P (%)		K (%)	
	1st Season	2nd Season	1st Season	2nd Season	1st Season	2nd Season
SPF1	1.48 ± 0.04 bc	1.36 ± 0.03 d	0.66 ± 0.02 abc	0.84 ± 0.03 ab	4.31 ± 0.18 ab	4.45 ± 0.06 b
SPF2	1.81 ± 0.03 a	1.91 ± 0.03 a	0.70 ± 0.03 a	0.88 ± 0.04 a	4.81 ± 0.35 a	4.99 ± 0.01 a
SPW	1.27 ± 0.08 cd	1.17 ± 0.08 e	0.63 ± 0.02 c	0.81 ± 0.02 ab	4.07 ± 0.04 ab	4.16 ± 0.01 c
FSF1	1.43 ± 0.10 bc	1.53 ± 0.04 c	0.64 ± 0.02 bc	0.81 ± 0.02 ab	4.23 ± 0.17 ab	4.39 ± 0.09 b
FSF2	1.53 ± 0.02 b	1.75 ± 0.04 b	0.69 ± 0.02 ab	0.83 ± 0.02 ab	4.29 ± 0.10 ab	4.44 ± 0.09 b
FSW	1.19 ± 0.04 d	1.10 ± 0.04 e	0.62 ± 0.01 c	0.72 ± 0.06 b	3.81 ± 0.29 b	3.98 ± 0.13 c

Mean values sharing the same lowercase letters (a–e) in the same column do not differ significantly at *p* ≤ 0.05 by Duncan’s multiple tests. SPF1; seed priming with 1.5 g L^−1^ fulvic acid, SPF2; seed priming with 3 g L^−1^ fulvic acid, SPW; seed priming with distilled water, FSF1; foliar spray with 1.5 g L^−1^, FSF2; foliar spray with 3 g L^−1^ fulvic acid, and FSW; foliar spray with tap water.

## Data Availability

The original contributions presented in the study are included in the article; further inquiries can be directed to the corresponding author.
